# Children’s Interstitial and Diffuse Lung Diseases (ChILD) in 2020

**DOI:** 10.3390/children7120280

**Published:** 2020-12-09

**Authors:** Valentina Agnese Ferraro, Stefania Zanconato, Andrea Zamunaro, Silvia Carraro

**Affiliations:** Pediatric Respiratory Medicine and Allergy Unit, Women’s and Children’s Health Department, University of Padova, via Giustiniani 2, 35128 Padova, Italy; stefania.zanconato@aopd.veneto.it (S.Z.); andrea.zamunaro@gmail.com (A.Z.); silvia.carraro@unipd.it (S.C.)

**Keywords:** children interstitial lung disease (chILD), chest high resolution computed tomography (HRCT), genetic tests

## Abstract

The term children interstitial lung diseases (chILD) refers to a heterogeneous group of rare diseases that diffusely affect the lung. ChILD specific to children younger than 2 years of age include diffuse developmental disorders, growth abnormalities, specific conditions of undefined etiology (neuroendocrine cell hyperplasia of infancy and pulmonary interstitial glycogenosis) and surfactant protein disorders. Clinical manifestations are highly variable, ranging from the absence of relevant symptoms to a severe onset. Most commonly, chILD presents with nonspecific respiratory signs and symptoms, such as dyspnea, polypnea, dry cough, wheezing, recurrent respiratory infections and exercise intolerance. In the diagnostic approach to a child with suspected ILD, chest high resolution computed tomography and genetic tests play a central role. Then, if the diagnosis remains uncertain, laryngotracheal-bronchoscopy and lung biopsy are needed. Pharmacological treatment is mostly empiric and based on anti-inflammatory and immunomodulatory drugs including corticosteroids, hydroxychloroquine and azithromycin. Despite chILD overall rarity, pediatric pulmonologists must be familiar with these diseases in order to carry out a timely diagnosis and patient treatment.

## 1. Introduction

ChILD (children interstitial lung disease, chILD) is a heterogeneous group of rare, chronic respiratory diseases, with a prevalence variably reported (from 0.13 to 16.2 per 100,000 children per year) as a result of a diagnosis which is often challenging [1]. In fact, standardized diagnostic criteria are lacking and the presentation clinical and pathological is heterogeneous.

Even if the term of Children interstitial lung disease, chILD, is conventionally used in the international scientific literature, the term Diffuse Lung Disease, DLD, would be more appropriate to describe these conditions, since many of them do not show exclusive involvement of the interstitium but also alveoli, distal small airways and/or terminal bronchioles [2]. In this manuscript, we use the term chILD meaning diffuse lung disease in children.

## 2. Classification

Different classification systems for chILD have been proposed in the past decade [3]. In 2004 Clement et al. [4] proposed the first chILD classification, based on etiology and physiopathology. This classification was extended by Deutsch et al. in 2007 [5] on the base of lung biopsies collected in children less than 2 years of age. Later, a further sub-classification has been introduced, separating ILD specific to infancy from other pediatric ILD, again on the base of etiologic and pathologic criteria [6,7,8,9]. According to Rice et al. [7], chILD can be classified as detailed in Table 1. In our review we will focus on chILD disorders more prevalent in infancy.

## 3. Clinical Manifestation

The severity of chILD presentation is highly variable, ranging from mild nonspecific symptoms, which my lead to a late diagnosis, to a very severe clinical picture. Usually the earlier is the onset of the disease, the more severe are the presenting symptoms. Despite the heterogeneity that characterizes chILD (age of presentation, genetic mutations, disease course), there is significant overlap in clinical manifestations [10].

The earliest possible presentation of chILD is shortly after birth, with unexplained respiratory distress in term neonates, who can rapidly require intubation and ventilation [11]. Less frequently, chILD patients are born preterm; in this case they present with an acute respiratory distress which is more severe than would be expected because of prematurity [11].

During the first two years of life [1,12], chILD clinical manifestations range from no symptoms to severe respiratory distress usually triggered by viral infections. In most cases, children with chILD have nonspecific respiratory signs and symptoms, such as dyspnea, polypnea, dry cough, wheezing, recurrent respiratory infections and exercise intolerance [11].

Older children can show tachypnoea, hypoxia, digital clubbing, and/or cyanosis during exercise or at rest [3,6,11].

In chILD auscultation typically reveals crackles, sometimes coupled with wheezes, but no pathological chest sound can be heard in up to a third of affected children [11].

## 4. Diagnosis

The diagnostic approach to a term neonate with respiratory distress of unknown origin or to an infant/child with persistent tachypnoea, cough, hypoxemia and diffuse pulmonary infiltrates, requires, as a first step, the exclusion of less rare causes of diffuse lung diseases such as cystic fibrosis, congenital cardiac diseases, primary ciliary dyskinesia, immunodeficiency, infections and recurrent aspiration [13]. Once major non-chILD disorders have been excluded, a “chILD syndrome” can be suspected in the presence of at least 3 out of the following 4 criteria [1,8,11,13]:

- respiratory symptoms (cough, rapid breathing, or exercise intolerance);
- respiratory signs (resting tachypnoea, auscultation with pathological sounds even if child has no acute infection, retractions, digital clubbing, respiratory failure, failure to thrive);
- hypoxemia;
- diffuse abnormalities on chest imaging.

**Figure 1:**
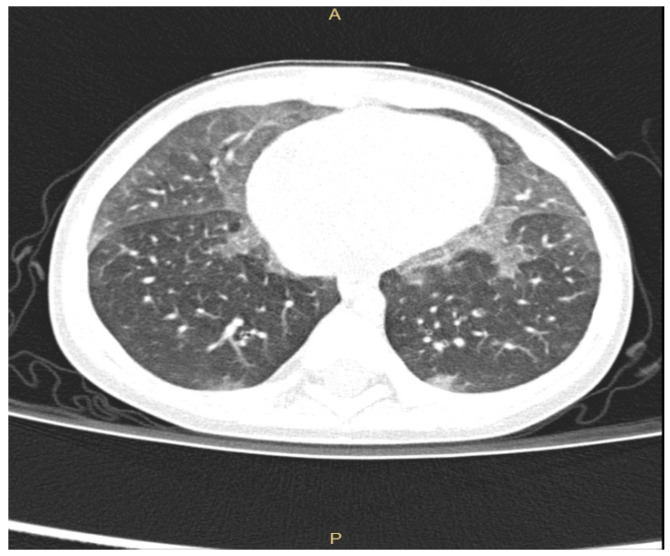
ground-glass opacities located in middle lobe and lingula in a 10-month-old infant with NEHI.

Even if plain radiographs are usually performed in a child suspected of ILD, the information provided is often limited. Thus, chest High Resolution Computed Tomography (HRCT) is the gold standard to investigate lung damage in these children [6,11,13], enabling the visualization of the parenchymal structure to the level of secondary pulmonary lobule. Furthermore, chest HRCT gives accurate information about the pattern of parenchymal abnormalities as well as about their extension and distribution. Common radiologic patterns in chILD are widespread ground-glass attenuation sometimes coupled with intralobular lines, irregular interlobular septal thickening, honeycombing and, less frequently, large subpleural air cysts (usually located in upper lobes adjacent to areas of ground-glass opacities) [6,14,15,16]. HRCT has indeed very poor sensitivity and specificity for the discrimination between chILDs, with the exception of neuroendocrine cell hyperplasia of infancy (NEHI), for which HRCT has 78% specificity and 100% sensitivity [17]. NEHI shows in fact a typical HRCT pattern characterized by ground-glass opacities located in middle lobe and lingula, as shown in Figure 1. In children with surfactant protein disorders, chest HRCT shows diffuse ground-glass opacity and reticulation, but no data are presently available on sensitivity and specificity of these aspects [1].

The correct timing to perform chest HRCT in children with suspected ILD is nowadays still debated. Even if radiation risk restricts the use of chest HRCT in pediatric population, this risk needs to be balanced against the benefits of a prompt diagnosis, which could help clinician in this rare disease management. Furthermore, we have to keep in mind that younger children, neonates and infants have to be sedated during the acquisition of radiological images and this could impair lung ventilation leading to a wrong interpretation of lung images.

Within the diagnostic workup in a child with suspected ILD, beside lung imaging, genetic tests are gaining more and more importance for the identification of a specific chILD, as showed in Table 2 [18]. Each child with suspected ILD should perform genetic tests early in the diagnostic workup and the results should be promptly provided, since they can potentially clarify the diagnosis and guide the therapeutic approach.

When both lung imaging and genetic tests cannot lead to the formulation of a specific diagnosis, more invasive procedures are needed, such as laryngotracheal bronchoscopy (LTBS) and/or lung biopsy. LTBS enables the assessment of airway anatomical structure and the collection of samples from the distal airways (bronchoalveolar lavage [BAL], bronchial brushing, bronchial biopsy) for pathological and microbiological examination [19,20]. Lung biopsy is the last step in the diagnostic approach to a child with suspected ILD, but sometimes it is the only test that leads to a definitive etiologic diagnosis [7]. Although, as discussed below, the histological evaluation does not modify the global approach to chILD, which is mostly empiric and based on clinical experience, recent data suggest that it can affect the patient management, justifying empiric therapy or narrowing treatment modality [21]. The site of biopsy should be established by a multidisciplinary team (including surgeon, pathologist and pediatric pulmonologist) and based on the analysis of lung abnormalities distribution on a recent chest HRCT [11,22]. Biopsy should be preferably performed from two sites by an experienced surgeon, in order to avoid diagnostic errors or not suitable samples [7].

**Figure 2:**
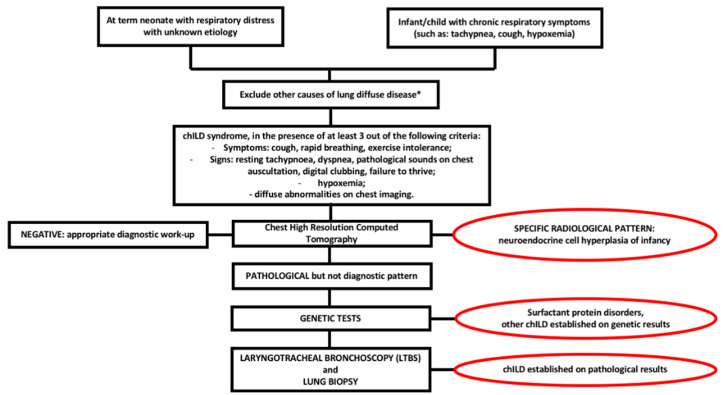
chILD diagnostic flow-chart. * cystic fibrosis, congenital heart diseases, primary ciliary dyskinesia, immunodeficit, infections, recurrent aspiration.

Figure 2 shows diagnostic flow-chart in child with suspected ILD.

## 5. Follow-Up and Prognosis

A multidisciplinary approach is mandatory in chILD, not only for diagnosis but also for follow-up. In this scenario the pediatric pulmonologist plays a key role, supported by the radiologist, the geneticist, the pathologist and the immunologist [1]. Also, the collaboration between pediatric pulmonologist and adult ILD specialist is important in view of an optimal management of children with ILD who survive beyond childhood. No standardized rules are nowadays available for follow-up in chILD, mostly because it depends on the specific chILD etiology, on patient clinical conditions, on number and severity of exacerbations.

The prognosis is heterogeneous (Table 3), ranging from complete recover in NEHI and pulmonary interstitial glycogenosis to a mortality rate that approaches 100% in alveolar capillary dysplasia. Cunningham et al., in a recent prospective cohort study including 127 children with chILD registered in the chILD EU registry, reported the following factors as being associated with poor prognosis: developmental/surfactant disorders, age at baseline (enrolment date) younger than 6 months, SpO2 at baseline <94% [23].

## 6. Therapeutic Approach

The treatment of patients with chILD is mainly supportive and based on oxygen supplementation and/or ventilation, adequate caloric intake and respiratory physiotherapy [1,10,11]. Oxygen and/or ventilation can be essential during the first days of life, then, depending on the course of the disease, they may be reduced and/or interrupted.

Pharmacological treatment is mostly empiric [1,33] and based on anti-inflammatory and immunomodulatory drugs including corticosteroids, hydroxychloroquine and azithromycin. Furthermore, lung transplant is a possible therapeutic option for patients with terminal chronic respiratory failure.

To go beyond an empiric approach and rely, instead, on an evidence-based approach, clinical trials exploring the efficacy and safety of pharmacological treatments in children with ILD are urgently needed.

Corticosteroids are effective in children with inflammatory lung damage, while they have limited impact in chILD without inflammation, such as NEHI. In ventilated patients it is suggested to use intravenous pulse methylprednisolone (10 mg/kg, even though some centers use 30 mg/kg), while in not ventilated patients oral prednisolone (2 mg/kg) can be an alternative [11].

Hydroxychloroquine is an immunosuppressant inhibiting T-cell function and, even if no randomized controlled trials are available (11), a good response to hydroxychloroquine has been reported in some cases of SFTPC and ABCA3 mutations [34,35,36]. *Griese* et al. have recently proposed an exploratory, Phase 2a, randomized, double-blind, placebo-controlled, multinational study investigating the impact of hydroxychloroquine in subjects with chILD [37].

Azithromycin has antibiotic, anti-inflammatory and immunomodulatory effect, but its own therapeutic effect is difficult to analyze, because in chILD azithromycin is always used in combination with corticosteroids and/or hydroxychloroquine [1].

Emerging therapeutic approaches for chILD include primary genetic correction and the use of stem cells and anti-fibrotic agents [1]. Pre-clinical studies investigated gene correction in hereditary pulmonary alveolar proteinosis with CSF2RA mutations [38] and in surfactant protein B deficiency [39], while stem cells have been used to correct pulmonary alveolar proteinosis in CSF2RB deficient mice models [40]. Antifibrotics (such as pirfenidone and nintedanib) are nowadays approved in adult idiopathic pulmonary fibrosis, being able to slow the rate of decline in forced vital capacity [1]. Even if pulmonary fibrosis is described only in a small proportion of chILD, it seems that, unlike in adults, children have few fibrosis lesions with more cellular recruitment, less collagen deposition and less parenchymal destruction, which allow for considering the significant beneficial effects of antifibrotic therapies [41]. Studies to extend the use of these therapeutic agents in children are now ongoing, as the first chILD clinical trial on Nintedanib for fibrotic diffuse lung disease in children [42].

## 7. Conclusions

ChILD is a heterogeneous group of rare, chronic respiratory diseases, affecting not only the insterstitium but also alveoli, distal small airways and/or terminal bronchioles. The high variability of chILD clinical manifestations, which range from mild symptoms to a severe onset, leads to a difficult and often delayed diagnosis and treatment. Despite their overall rarity, pediatric pulmonologists must be familiar with these diseases in order to carry out a timely diagnosis and patient management.

## Figures and Tables

**Table 1:** chILD classification according to Rice et al. [7].

**DISORDERS MORE PREVALENT IN INFANCY**
**Diffuse developmental disorders:** - acinar dysplasia - alveolar capillary dysplasia - congenital alveolar dysplasia
**Growth abnormalities:** - alveolar hypoplasia - chronic neonatal lung disease - related to chromosomal disorders - related to congenital heart disease
**Specific conditions of undefined aetiology:** - neuroendocrine cell hyperplasia of infancy (NEHI) - pulmonary interstitial glycogenosis (PIG)
**Surfactant protein disorders**
**DISORDERS RELATED TO SYSTEMIC DISEASE PROCESSES**
**Disorders related to systemic disease:** - Langerhans cell histiocytosis - related to acquired heart disease - storage disease/endogenous lipid pneumonia
**Disorders of the normal host (presumed immune intact):** - eosinophilic bronchiolitis/pneumoniae - infection/post infectious processes - hypersensitivity pneumonitis - aspiration pneumonia
**Host disorders (immunocompromised):** - opportunistic infection - transplant-related
**Disorders masquerading as ILD:** - pulmonary hypertension - veno-occlusive disease - lymphatic disorders - capillary haemangiomatosis - thromboembolic disease - vasculitis
**Lymphoproliferative disease:** - lymphoid interstitial pneumonia - diffuse lymphoid hyperplasia - lymphomatoid granulomatosis
**Small airways disease:** - chronic bronchiolitis - obliterative bronchiolitis - follicular bronchiolitis
**Interstitial pneumonias unrelated to surfactant protein disorder:** - organizing pneumonia - diffuse alveolar damage> - usual interstitial pneumonia
**Other patterns of diffuse lung disease:** - hemosiderosis - alveolar microlithiasis - sarcoidosis

**Table 2:** Genetic mutations associated with chILD.

Genetic Mutation	Inheritance	chILD
ABCA3 (ATP-binding cassette-family A-member 3)	Autosomal recessive	Surfactant protein disorder
COPA (coatomer associated protein subunit alpha)	Autosomal dominant	General disorder, involving lung, joint, kidney
CSF2RA (colony stimulating factor 2 receptor α)	X-linked	Pulmonary alveolar proteinosis
CSF2RB (colony stimulating factor 2 receptor β)	Autosomal recessive	Pulmonary alveolar proteinosis
FLNA (Filamin A)	X-linked recessive	General disorder, involving lung, cardiovascular system, osteo-articular system, coagulation
FOXF1 (forkhead box F1)	Autosomal dominant	Diffuse developmental disorders, such as alveolar capillary dysplasia withmisalignment of the pulmonary veins
GATA2 (GATA Binding Protein 2)	Autosomal dominant	Immunodeficiency 21 with pulmonary alveolar proteinosis
MARS (metionil-transfer RNA sintetasi)	Autosomal recessive	Pulmonary alveolar proteinosis
NKX2-1 (NK2 homeobox 1)	Autosomal dominant	Brain lung thyroid syndrome, characterized by congenital hypothyroidism, hypotonia, chorea, interstitial lung disease
NSMCE3 (Non-structural maintenance of chromosomes element 3 homolog)	Autosomal recessive	Immunodeficiency
OAS1 (oligoadenylate synthetase 1)	Autosomal dominant	Pulmonary alveolar proteinosis with hypogammaglobulinemia and splenomegaly
SFTPB (Surfactant protein B deficiency)	Autosomal recessive	Surfactant protein disorder
SFTPC (Surfactant protein C mutation)	Autosomal dominant	Surfactant protein disorder
SLC7A7 (solute carrier family 7 member 7)	Autosomal recessive	Pulmonary alveolar proteinosis with lysinuric protein intolerance
TBX4 (T-box transcription factor 4)	Autosomal dominant	Acinar dysplasia
TMEM173 (transmembrane protein 173)	Autosomal dominant	Lung fibrosis with general inflammation

**Table 3:** Clinical presentation and prognosis of chILD.

chILD		Clinical Presentation and Prognosis
Diffuse developmental disorders	Acinar dysplasia	Cyanosis at birth, survival only for few hours [24]
	Congenital alveolar dysplasia	Variable clinical presentation and prognosis [24]
	Alveolar capillary dysplasia	Onset in first 24 h of life, death within days/weeks after presentation [25]. Less frequently onset during the first months of life [24].
Growth abnormalities	Alveolar hypoplasia	Variable clinical presentation and prognosis
	Bronchodysplasia	Variable clinical presentation and prognosis
	Related to chromosomal disorders	Variable clinical presentation and prognosis, related to the specific chromosomopathy
	Related to congenital heart disease	Variable clinical presentation and prognosis, related to the specific congenital heart disease
Specific conditions of undefined aetiology	Neuroendocrine cell hyperplasia of infancy	Onset during the first months of life, variable symptoms thereafter; no deaths and no need of lung transplant described [26,27]
	Pulmonary interstitial glycogenosis	Onset immediately after birth. Good prognosis without severe comorbidities, but respiratory symptoms can persist into adolescence [28]
Surfactant protein disorder	SPFTB mutations	Neonatal onset with poor prognosis, even if rarely patients can survive longer [29]
	SPFTC mutations	Variable clinical presentation and prognosis [30]
	ABCA3 mutations	Neonatal onset with poor prognosis (or, less frequently, presentation at an older age and longer survival) [31,32]
